# Comorbidities and healthcare costs and resource use of patients with nonalcoholic fatty liver disease (NAFLD) and nonalcoholic steatohepatitis (NASH) in the Japan medical data vision database

**DOI:** 10.1007/s00535-021-01759-2

**Published:** 2021-01-26

**Authors:** Shuji Terai, Amy Buchanan-Hughes, Alvin Ng, I-Heng Lee, Ken Hasegawa

**Affiliations:** 1 Division of Gastroenterology and Hepatology, Graduate School of Medical and Dental Sciences, Niigata University, Niigata, Japan; 2 Costello Medical, Boston, MA USA; 3 Costello Medical, Singapore, Singapore; 4 Gilead Sciences Inc, Foster City, CA USA

**Keywords:** Cost, Database analysis, Japan, Nonalcoholic fatty liver disease, Nonalcoholic steatohepatitis

## Abstract

**Background:**

This study examined demographics, comorbidities and healthcare resource use (HCRU) and costs among Japanese patients with nonalcoholic fatty liver disease (NAFLD) and nonalcoholic steatohepatitis (NASH).

**Methods:**

We conducted a repeated cross-sectional analysis of the Medical Data Vision (MDV) claims database, from January 2011 to March 2018. Demographics were described at index date and by calendar year; a “NASH” subpopulation included patients with ≥ 1 claim for NASH at any time. Prevalence of pre-specified comorbidities of interest and data-emergent top comorbidities were estimated. All-cause HCRU and costs were quantified by calendar year. Outcomes were compared between 2011 and 2017 using partially overlapping *t* tests.

**Results:**

58,958 patients (mean age 61.6 years; 55.5% male) were included. 1139 patients (2%) were in the NASH subpopulation. At baseline, comorbid cardiovascular disease (69.4%), diabetes (62.1%) and hyperlipidaemia (54.4%) were most prevalent; comorbidity prevalence increased with age. Mean outpatient visits decreased from 9.36 per patient in 2011 to 7.80 in 2017; mean inpatient admissions increased (both *p* < 0.001 for 2011 vs 2017). Mean total all-cause healthcare costs ranged from ¥322,206 to ¥340,399 per patient per year between 2011 and 2017. Although total all-cause healthcare costs did not change significantly (*p* = 0.552), cost burden shifted from the outpatient to inpatient setting between 2011 and 2017. All-cause healthcare resource use/costs were generally higher for the NASH subgroup compared with the overall population.

**Conclusions:**

There is a high burden of disease among Japanese NAFLD/NASH patients, including a high prevalence of comorbidities which generally increase with age. Accordingly, substantial all-cause HCRU and costs were incurred.

**Supplementary Information:**

The online version contains supplementary material available at 10.1007/s00535-021-01759-2.

## Introduction

Nonalcoholic fatty liver disease (NAFLD) is characterised by the accumulation of fat in liver cells. It is recognised as the most common cause of chronic liver disease in Asian and Western countries, and its prevalence has been increasing rapidly in Asia–Pacific in recent years [1, 2]. Nonalcoholic steatohepatitis (NASH) is a more serious, clinically progressive form of inflammatory NAFLD, and can lead to fibrosis, cirrhosis, and hepatocellular carcinoma (HCC) [3].

Although NAFLD/NASH is strongly correlated with obesity [4], there is a considerable number of patients with “lean” NAFLD/NASH; the prevalence of NAFLD in the non-obese population (body mass index [BMI] < 25 kg/m^2^) may be higher in Asia (7–19%, 15% in Japan) compared with Western countries (~ 10%) [4, 5].

NAFLD/NASH is associated with several metabolic comorbidities, including diabetes mellitus (DM), hyperlipidaemia and dyslipidaemia, and is also correlated with increased risk of cardiovascular disease (CVD) and chronic kidney disease (CKD) [6, 7]. The management of these conditions can substantially add to the burden of disease, and may complicate the treatment of NAFLD/NASH, impacting clinical care outcomes [6, 8, 9]. The prevalence of NAFLD/NASH is generally higher in males and in older age, which may be attributable to differences in the prevalence of obesity and metabolic syndrome [3, 6, 10]. The proportion of individuals with NASH among the NAFLD population is forecasted to increase in the coming decades due to an ageing population and the projected rising prevalence of DM worldwide [11].

In Japan, based on a cross-sectional study of three health centres in 2009–2010, the prevalence of NAFLD among 5,075 patients attending a health check, who did not have markers of hepatitis B infection, hepatitis C infection, or alcoholic liver disease, was 29.7% [10]. A later Markov model estimated the prevalence of NAFLD in the general population in Japan at 17.9% in 2016, of which 16.6% had NASH (3% of the general population) [11]. While the current rate of liver-related complications from NAFLD appears low based on limited available data, studies have noted a rising trend [4], and the number of NAFLD with compensated cirrhosis and end-stage disease in Japan is predicted to increase by 64% from 2016 to 2030 (to 282,670 prevalent cases from 127,840) [11].

Studies of health resource use and cost in the US and the EU have estimated substantial, rising clinical and economic burdens of NAFLD [11–14]. Advanced liver disease such as decompensated cirrhosis is especially cost- and resource-intensive [11]. Yet, with no currently approved treatments for NASH, there is a lack of attention on the unmet needs of the NAFLD/NASH patient population [15]. The direct costs of nonalcoholic liver cirrhosis, including viral hepatitis-related cirrhosis, in Japan was estimated at 29.0 billion yen in 2014 [16]; an ongoing need exists to better quantify the NAFLD and NASH-related burden in Japan, including estimations of health resource use and costs.

### Objectives

This study aimed to examine demographics, comorbidities and healthcare resource costs and use among Japanese patients with NAFLD and NASH via a retrospective repeated cross-sectional analysis of the Medical Data Vision (MDV) claims database.

## Methods

### Data source

This retrospective repeated cross-sectional observational study used data from the Japan MDV database. MDV contains electronic health insurance claims and Diagnosis Procedure Combination (DPC) payment system data from over 300 Japanese acute hospitals, covering over 22 million patients as of March 2018. Anonymised information is available on patient demographics, laboratory values, medical procedures, disease diagnoses, and inpatient and outpatient resource use and costs. This study used data from January 2011 to March 2018, inclusive.

### Study population and eligibility criteria

In the MDV, claims are tagged with standard disease code “DiseaseCode” values which are specific to Japan, each of which has a corresponding “DiseaseName” describing the disease associated with the claim. Each DiseaseCode is also mapped to 4-character International Classification of Disease Version 10 (ICD-10) codes. To be eligible for this study, patients had to have at least one claim for NASH (DiseaseCode: 8,843,497, “nonalcoholic steatohepatitis”, mapped to ICD-10 code: K75.8) or NAFLD (DiseaseCode: 5,718,008, “hepatic steatosis”, mapped to ICD-10 code: K76.0) between January 2011 and March 2018 inclusive. Patients had to be aged ≥ 18 years at index date, have at least six months enrolment (≥ 1 claim for any disease, treatment or procedure) pre-index date and at least three months’ enrolment post-index date. The index date was defined as the first day of the month of the patient’s first visit for NASH or NAFLD where they were ≥ 18 years old.

Exclusion criteria were claims for any of the following conditions at any time: hepatitis B, C or other viral hepatitis; HIV; alcohol-related conditions (including acute alcoholism, alcoholic gastritis, and alcoholic fatty liver); toxic liver disease; Wilson’s disease; autoimmune hepatitis; Gaucher or liposomal acid lipase deficiency (LAL-D); biliary cirrhosis; cholangitis; hemochromatosis. ICD-10 and standard disease codes used to identify patients are listed in Online Resource 1.

As an exploratory analysis, we also conducted analyses in a “NASH” subpopulation, defined as patients with at least one claim for NASH, with or without NAFLD, at any time during the study period.

### Statistical analysis

Demographic characteristics (age and gender) were described for the overall NAFLD/NASH study population at index date, as well as for each calendar year from 2011 to 2017 for patients who had made at least one claim in the relevant year. Age was obtained at the patients’ index claim, or at their first claim within the calendar year for the calendar year analysis. BMI was calculated from the height and weight data recorded during the closest available month within the baseline period to the NAFLD/NASH index date, from patients with available height and weight data (which are only recorded after discharge from an inpatient stay). The percentage of patients with non-obese (BMI < 25) NAFLD/NASH at baseline was summarised based on Asia–Pacific obesity classification criteria [17]: underweight (< 18.5 kg/m^2^), normal weight (18.5–22.9 kg/m^2^), overweight/at risk (23.0–24.9 kg/m^2^), obese I (25.0–29.9 kg/m^2^), and obese II (≥ 30 kg/m^2^). “Lean” NAFLD/NASH is defined as having a BMI of < 23 [17].

A one-way chi-squared test was used to compare the gender distribution in the study population with the overall Japanese population. Pearson’s chi-squared and one-way ANOVA tests were conducted to gauge whether the distributions of age and gender changed year-on-year between 2011 and 2017 for patients with index dates in each year.

A patient was defined as having a comorbidity if they had at least one claim for the relevant ICD-10 code within 6 months before to 3 months after the NAFLD/NASH index date. Seven comorbidity categories of interest identified with ICD-10 diagnosis codes (Online Resource 2) were pre-specified: CVD; hypertension; hyperlipidaemia; renal impairment, and subgroup chronic kidney disease/end-stage renal disease (CKD/ESRD); diabetes, and subgroup type 2 diabetes (T2D). The prevalence of these pre-defined comorbidity groups was reported for the overall NAFLD/NASH population.

A separate analysis was conducted to determine data-emergent comorbidities in the population, to explore any prevalent comorbidities that may have been missed under the pre-specified categories. Using the first three characters of ICD-10 codes (e.g. E78.X) [18], which was deemed a reasonable approach to define diagnosis categories that were neither too broad nor too specific, the prevalence of the top 10 most common comorbidities were reported. The prevalence of the top 10 data-emergent comorbidities as well as the eight pre-specified comorbidity categories of interest listed above were stratified by age groups. Associations between age and each comorbidity/comorbidity group were tested with Pearson’s chi-squared tests.

All-cause healthcare resource use was quantified by the number of outpatient visits, as well as by the number and duration of inpatient visits. All-cause healthcare costs included the costs of inpatient and outpatient visits, laboratory tests, and drug use. Summary statistics (mean, standard deviation, 95% CI, minimum, maximum, median, Q1 and Q3) were produced for each resource use or cost item by calendar year. As the samples were neither totally independent nor totally dependent between calendar years, partially overlapping t tests were conducted to compare mean healthcare resource use and cost items between 2011 and 2017 [19, 20].

Since many statistical tests were performed across the study, the False Discovery Rate (FDR) method (suggested by Benjamini and Hochberg) was applied to correct for multiple testing [21, 22]. The threshold for statistical significance of the adjusted *p*-values was 0.05. All analyses were independently dual-programmed using SAS version 9.4 and R version 3.5.3; the outputs from each step were compared and any discrepancies were discussed and resolved. When necessary, a third reviewer was available to assist with troubleshooting and to ensure alignment of the approach with the analysis plan.

## Results

### Patient disposition and baseline characteristics

**Fig. 1:**
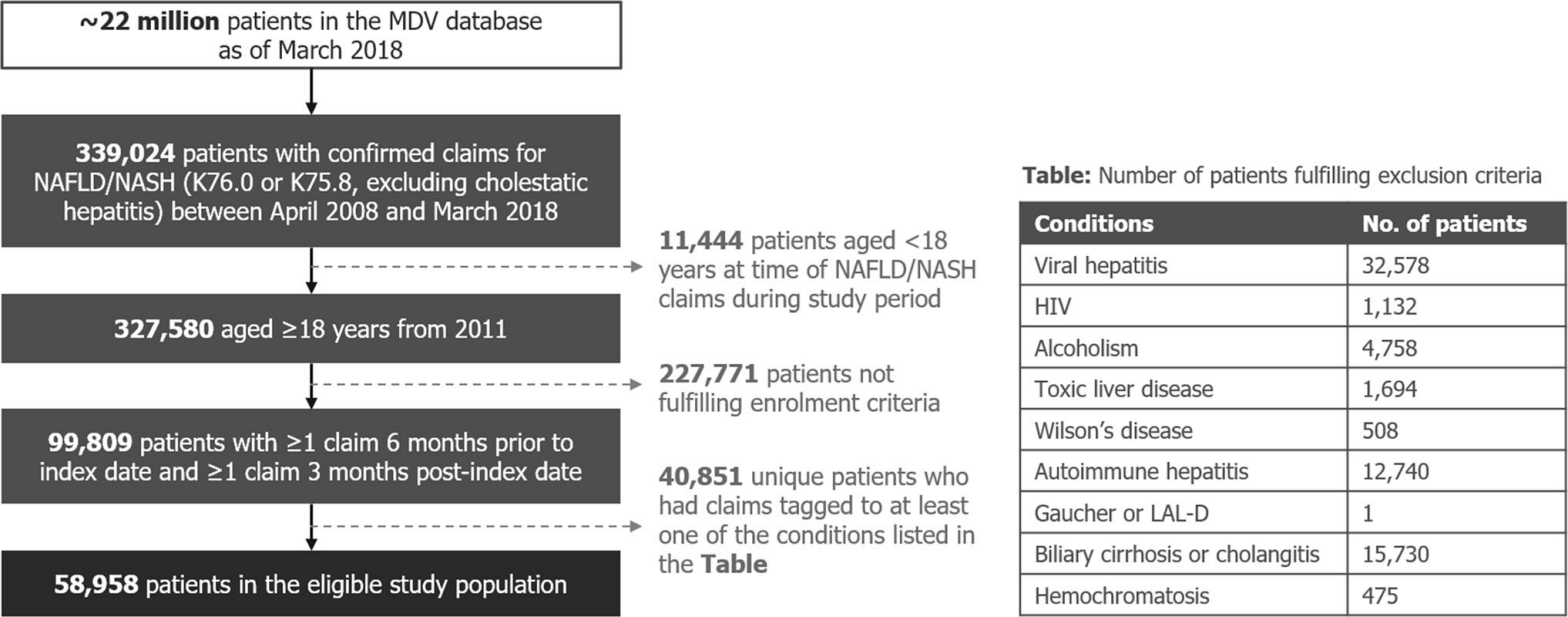
MDV database eligible sample flowchart. *HIV* human immunodeficiency virus, *LAL-D* lysosomal acid lipase deficiency, *MDV* Medical Data Vision, *NAFLD* nonalcoholic fatty liver disease, *NASH* nonalcoholic steatohepatitis

Overall, there were 58,958 patients with records in the MDV database from January 2011 to December 2017 who fulfilled the inclusion criteria (Fig. 1). The mean age of the overall study cohort was 61.6 years at index date, with the mean age of each calendar year study cohort increasing slightly over each subsequent year (Table 1). A slight majority (55.5%) of included patients were male; a one-way chi-squared test showed a significant difference (*p* < 0.001) comparing against the gender distribution of the general population as reported in the 2015 Japanese Census [23]. Descriptive statistics for the calendar year analysis comparing cohorts based on patients’ index dates can be found in Online Resource 3, Supplementary Table 1; demographic characteristics for the NASH subpopulation can be found in Online Resource 3, Supplementary Table 2.

**Table 1:** Demographic characteristics of overall population, and stratified by year

Parameter	Variable	At index date	2011	2012	2013	2014	2015	2016	2017
		(*N* = 58,958)	(*N* = 11,515)	(*N* = 14,030)	(*N* = 17,485)	(*N* = 23,086)	(*N* = 31,212)	(*N *= 40,399)	(*N* = 48,364)
		*n* (%)	*n* (%)	*n* (%)	*n* (%)	*n* (%)	*n* (%)	*n* (%)	*n* (%)
Gender	Male	32,698 (55.5)	6,429 (55.8)	7,850 (56.0)	9,732 (55.7)	12,859 (55.7)	17,314 (55.5)	22,313 (55.2)	26,548 (54.9)
	Female	26,260 (44.5)	5,086 (44.2)	6,180 (44.1)	7,753 (44.3)	10,227 (44.3)	13,898 (44.5)	18,086 (44.8)	21,816 (45.1)
Age group, years^a^	18–34	2,409 (4.1)	264 (2.3)	340 (2.4)	460 (2.6)	662 (2.9)	979 (3.1)	1,250 (3.1)	1,472 (3.0)
	35–44	5,293 (9.0)	823 (7.1)	1,002 (7.1)	1,268 (7.3)	1,693 (7.3)	2,283 (7.3)	2,962 (7.3)	3,365 (7.0)
	45–54	8,897 (15.1)	1,529 (13.3)	1,796 (12.8)	2,240 (12.8)	2,978 (12.9)	4,173 (13.4)	5,438 (13.5)	6,529 (13.5)
	55–64	14,334 (24.3)	3,210 (27.9)	3,803 (27.1)	4,416 (25.3)	5,543 (24.0)	7,100 (22.8)	8,725 (21.6)	10,087 (20.9)
	65–74	17,434 (29.6)	3,410 (29.6)	4,148 (29.6)	5,318 (30.4)	7,233 (31.3)	9,914 (31.8)	13,054 (32.3)	15.471 (32.0)
	75 +	10,591 (18.0)	2,279 (19.8)	2,941 (21.0)	3,783 (21.6)	4,977 (21.6)	6,763 (21.7)	8,980 (22.2)	11,440 (23.7)
Age, years^a^	Mean (SD)	61.63 (13.99)	63.29 (12.93)	63.55 (13.09)	63.63 (13.28)	63.58 (13.48)	63.49 (13.67)	63.65 (13.72)	64.00 (13.76)
	Median (IQR)	64 (19)	64 (17)	65 (17)	65 (17)	65 (17)	65 (18)	66 (19)	66 (19)

^a^For the calendar year analyses, the age of patients in each calendar year cohort was obtained at their first claim within the calendar year. Patients who had at least one claim in a particular year were included in that year’s analysis

*IQR* interquartile range, *SD* standard deviation

Of 58,958 total patients, 1139 patients (2%) were included in the NASH subpopulation (i.e. had at least one claim for NASH at any time during the study). 9620 patients (16% of the eligible sample) had available data for height and weight within their baseline period; of these, 44.8% were non-obese, based on a calculated BMI of < 25; 25.4% had “lean” NAFLD/NASH (BMI < 23). Obesity rates were generally higher in younger age groups (75.0% in patients aged 18–34 vs 41.5% in patients aged 75 and over; Online Resource 3, Supplementary Table 3).

### Comorbidities

**Fig. 2:**
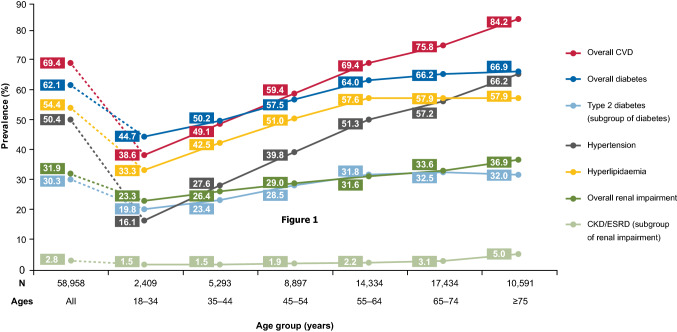
Prevalence of comorbidities of interest at baseline, stratified by age group. “Type 2 diabetes” is a subgroup of “Overall diabetes”; “CKD/ESRD” is a subgroup of “Overall renal impairment”. *CKD* chronic kidney disease, *CVD* cardiovascular disease, *ESRD* end-stage renal disease

Prevalence at baseline of the comorbidities of interest are summarised in Fig. 2. CVD was most prevalent in the overall study population (69.4%), followed by diabetes (62.1%; 30.3% with T2D). About half of included patients had hyperlipidaemia (54.4%) and hypertension (50.4%) at baseline. Less than a third of patients had renal impairment (31.9%), and only 2.8% had CKD or ESRD.

The prevalence of comorbidities at baseline generally increased with age for all the comorbidities of interest, with the lowest rates in those aged 18–34 years. This increase was especially stark for CVD, which was present in 38.6% of patients aged 18–34 years, rising to 84.2% of patients aged 75 and older, and hypertension, which had a prevalence of 16.1% in the youngest age group and 66.2% in the oldest (Fig. 2).

**Fig. 3:**
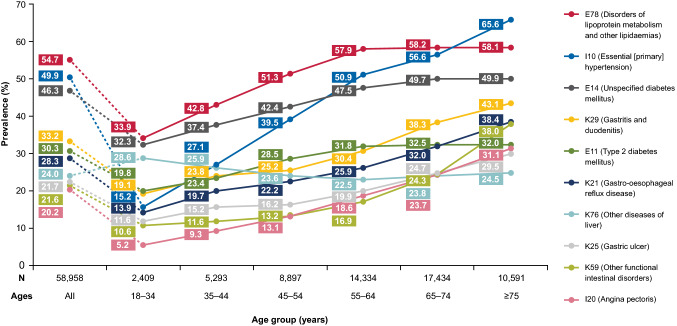
Prevalence of overall top 10 comorbidities at baseline across age groups. Comorbidity categories were defined according to ICD-10 descriptions

Emergent top 10 most prevalent comorbidities at baseline based on an analysis of the ICD-10 codes attached to claims were: disorders of lipoprotein metabolism and other lipidaemias (54.7%); primary hypertension (49.9%); unspecified DM (46.3%); gastritis and duodenitis (33.2%); T2D (30.3%); gastro-oesophageal reflux disease (28.3%); other diseases of liver (24.0%); gastric ulcer (21.7%); other functional intestinal disorders (21.6%); and angina pectoris (20.2%). When stratified by age, disorders of lipoprotein metabolism and other lipidaemias remained the top most prevalent comorbidity in every age group except those aged 75 above, amongst whom the prevalence of primary hypertension was highest (Fig. 3).

### All-cause healthcare resource use and costs

**Fig. 4:**
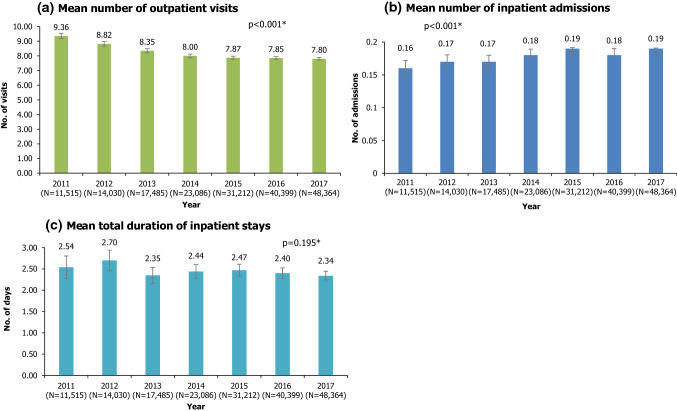
All-cause healthcare resource use, by calendar year. *P values from partially-overlapping *t*-test for comparison of means between 2011 and 2017. Error bars represent 95% confidence intervals. N is the number of patients with at least one claim in that calendar year

Among patients included in each calendar year cohort (i.e. with at least one claim in that calendar year), almost all (> 99%) had at least one outpatient visit in that year. The mean number of outpatient visits decreased over the years, from 9.36 per patient in 2011 to 7.80 in 2017 (p < 0.001 for 2011 vs 2017; Fig. 4a). In each calendar year, about 12–14% of patients with claims in that year had at least one inpatient admission. The mean number of inpatient admissions increased between 2011 and 2017; however, the magnitude of increase was small (0.16 in 2011 vs 0.19 in 2017, p < 0.001; Fig. 4b). There was no statistically significant change over time in the mean total duration of inpatient admissions (*p* = 0.195 for 2011 vs 2017; Fig. 4c).

**Fig. 5:**
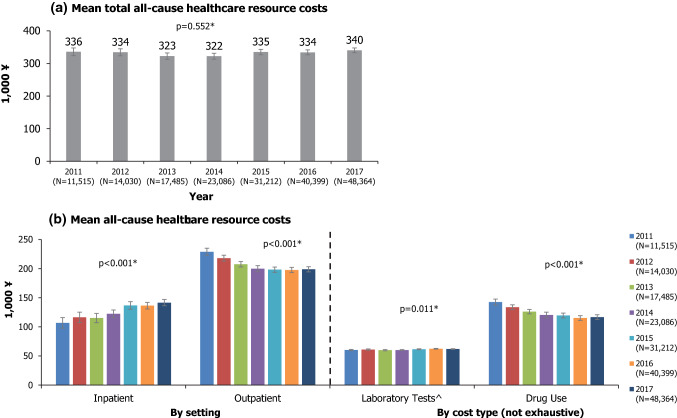
All-cause healthcare resource costs. *P values from partially overlapping t tests for comparison of means between 2011 and 2017. Error bars represent 95% confidence intervals. ^^^Includes costs from the test itself and materials that were used during the procedure (e.g. wound dressings)

Mean total all-cause healthcare costs ranged from ¥322,206–¥340,399 per patient per year between 2011 and 2017. Although the total all-cause healthcare costs did not change significantly between 2011 and 2017 (*p* = 0.552; Fig. 5a), the cost burden seemed to shift from the outpatient to inpatient setting across this time period (Fig. 5b). Mean drug use costs declined between 2011 and 2017 (¥142,580 vs ¥116,737; *p* < 0.001), while laboratory test costs, which include costs from the imaging or pathologic test itself and materials that were used during the procedure such as wound dressings, increased slightly from ¥60,245 in 2011 to ¥61,930 in 2017 (*p* = 0.011, Fig. 5b). Distributions for costs were highly right-skewed and means may have been inflated by outliers (e.g. a few patients with high claims); however, similar increasing or decreasing trends described above were seen when examining medians.

Mean number of inpatient admissions and duration per patient per year were generally higher for the NASH subpopulation compared with overall NAFLD/NASH population (Online Resource 3, Supplementary Fig. 1b and c). NASH patients had 0.31 mean admissions per year, staying for a mean of 4.10 days per year in 2017, while overall NAFLD/NASH patients had 0.19 mean admissions per year, staying an average of 2.34 days.

Mean all-cause total healthcare resource costs in the NASH subpopulation were also generally numerically higher than those in the overall NAFLD/NASH population across the years (Online Resource 3, Supplementary Fig. 2a; ¥442,723 vs ¥340,399 in 2017). This was driven both by generally higher inpatient and outpatient costs in the NASH subpopulation across all years, compared with the overall NAFLD/NASH population (Online Resource 3, Supplementary Fig. 2b and c). In terms of cost type, mean costs were numerically higher for the NASH subpopulation compared with overall NAFLD/NASH patients for both all-cause laboratory test costs (¥78,839 vs ¥61,930) and all-cause drug use costs (¥130,736 vs ¥116,737) in 2017.

## Discussion

### Patient characteristics and comorbidities

In this study, the proportion of male NAFLD/NASH patients was slightly higher than females. This aligns with previous data indicating an elevated prevalence of NAFLD/NASH in males, although gender differences in our study could have been attenuated by the older age distribution of included NAFLD/NASH patients, as while NAFLD is more common in men than in younger women, the prevalence of NAFLD is known to increase in post-menopausal women [10, 24].

A lower than expected proportion of NAFLD patients were identified as having progressed to NASH (2%), compared with previous estimates: a modelling study indicated a 16.6% prevalence of NASH among NAFLD patients in Japan [11]; in a cross-sectional study which identified NASH with advanced liver fibrosis, the prevalence among NAFLD patients was 8.3% based on a BAAT score of ≥ 3, and 2.7% based on a FIB-4 index of ≥ 2.67 [10]. The lower than expected NASH rates may have been due to a high proportion of misclassification errors in the billing data (i.e. claims for NASH patients continuing to only be recorded as NAFLD). Therefore, estimated differences between the NASH subgroup and the overall NAFLD/NASH population may have been diluted, even after accounting for up to 5% patients who may have been NASH regressors [11].

Preliminary analyses showed an unrealistically low prevalence (< 3%) of obesity based on claimed ICD-10 codes, indicating a high likelihood of underdiagnosis. Based on obesity rates as defined using BMI categories, which were only available for patients who were hospitalised, a quarter of NAFLD/NASH patients were non-obese, with this proportion of “lean” NAFLD/NASH patients increasing with increasing age. This concurs with the trend noted in other studies that a considerable proportion of NAFLD cases may occur in non-obese patients, particularly in Japan [5, 10] and other Asian countries [4, 25]. This reported higher prevalence of non-obese NAFLD patients compared with the Western population could be due to the higher proportion of PNPLA3 carriers in Japanese, a gene strongly associated with NAFLD progression [26].

There have been few population-based studies examining comorbidities among NAFLD/NASH patients in Japan [6]. Our database analysis found generally higher rates of comorbidities than have been previously reported: a retrospective cohort study of NAFLD patients at a Japanese public hospital found a 8.2% prevalence of diabetes (vs 62.1% in our study) [27], while the prevalence of hypertension among NAFLD patients in Japan has been previously estimated in the range of 12.9%–40.6% (vs 50.4% in our study) [6, 27, 28]. Elevated comorbidity rates could be due to a broad approach taken in the inclusion of code sets used to define each comorbidity group in our analysis (Online Resource 2).

In our analysis, the emergent top-most common comorbidities largely aligned with the pre-defined comorbidity categories of interest, with CVD, endocrine/nutritional/metabolic diseases, and diseases of the digestive system being most prevalent among patients with NAFLD/NASH in Japan across all age groups. This aligns with the findings of previous global studies, where metabolic disorders, CKD and CVD are well-known to be associated with NAFLD [6, 7]. NAFLD has been hypothesised to be on the causal pathway for CVD or CKD; however, the existence of an independent link is difficult to establish due to the sharing of multiple common risk factors between these conditions [6, 7]. Nevertheless, the high prevalence of comorbidities found among NAFLD/NASH patients in this study (particularly among older patients) implies an increased burden for clinical disease management [6, 29]; for instance, diabetes in NAFLD increases the risk of progression to cirrhosis and NASH [6], while cardiac-related death is one of the leading causes of mortality in NAFLD patients [3, 6, 24].

### Healthcare resource use and cost

Global modelling studies for NAFLD suggest a potentially growing clinical and economic burden of disease, owing to the increasing prevalence of obesity and metabolic disease worldwide [11, 13]. However, the increase in NAFLD cases was predicted to be lower in Japan due to declining population numbers [11].

In this study, outpatient healthcare resource use among NAFLD/NASH patients seemed to be decreasing over the years, although no clear trend was seen among NASH patients. Meanwhile, inpatient admissions and inpatient stay durations showed an increasing trend between 2011 to 2017, particularly among the NASH subgroup. As there are currently no approved therapies for the treatment of NAFLD or NASH, NAFLD/NASH-related healthcare costs include resources used in the management of liver complications and other comorbidities [13, 30]. Higher costs are particularly associated with progression to advanced liver disease, including the development of HCC [11]. The shifting burden from outpatient to inpatient resource use and costs could indicate an increasing proportion of patients progressing to more resource-intensive stages of disease, such as NASH with decompensated cirrhosis, or an increasing proportion of patients with serious comorbidities, in parallel with a generally ageing patient population.

With mean costs of up to ¥340,000 per patient per year, NAFLD/NASH patients in the MDV database incurred substantial all-cause healthcare costs, representing a significant burden to the healthcare system. This burden was greater for the subgroup of patients with claims for NASH (up to ¥495,000 per patient per year, or 30% higher than the mean for overall NAFLD/NASH patients in 2017).

The mean hospital-based costs for NAFLD/NASH patients incurred within a single institution tracked by this study were almost as high as the national average for health expenditures incurred across all settings—the Ministry of Health, Labor, and Welfare estimates that the per capita national medical care expenditure, which includes medical treatment, dental treatment, pharmacy dispensing, inpatient living and food expenses and nursing home-visits, but excludes expenditures not covered by health insurance, was ¥339,900 for 2017 [31] —suggesting that the total healthcare costs for patients within the spectrum of NAFLD/NASH, including advanced liver disease, are likely to be substantially higher than the general population in Japan.

### Limitations

There are limitations associated with the use of real-world claims data for epidemiological analysis; Alexander et al. found a large shortfall between the expected prevalence of NAFLD/NASH patients and the actual number recorded in European databases, due to possible under-diagnosis and under-recording [32]. The burden of NAFLD/NASH as quantified in this study may thus be conservative estimates of the actual disease burden in Japan.

The MDV database includes only hospitals under the DPC system, which is used by about 20% of total hospitals in Japan [33]. The study population thus represented only patients seeking care in acute care hospitals, and excluded those only managed in primary or specialist care settings, who may have different characteristics [34]. Furthermore, coverage of smaller-scale hospitals and clinics is limited [35]; thus, this study may have selected for a group of patients with more comorbidities compared with the wider NAFLD/NASH population.

Another limitation is the unavailability of biopsy information in the MDV database; therefore, only ICD-10 and standard disease codes could be used to define NAFLD/NASH in our study. The DiseaseCode “5,718,008, hepatic steatosis" that we used to identify NAFLD patients may include claims for alcoholic fatty liver disease; although we had excluded all patients with claims for alcohol-related conditions from the dataset, some patients with a history of alcohol abuse may nevertheless have been erroneously included. As height and weight data in the MDV were only available for patients who were discharged from an inpatient stay, which was a small percentage (16%) of the overall sample, findings on obesity may not necessarily be generalised to the wider NAFLD/NASH population. Furthermore, in our dataset, all patients over 90 years of age had their age recorded as “90” for all records; this might have truncated the upper end of the age distribution.

As the MDV does not offer linkage between institutions, resource use and cost estimates in this study are likely underestimations. Patients in Japan often visit multiple institutions for medical care [36]; if any patient visited healthcare institutions outside the coverage of MDV database or moved between institutions during the study, their resource use and costs in those visits would not be captured.

## Conclusions

Results from this analysis provide insights into the current disease landscape of NAFLD/NASH in Japan, particularly demonstrating the high burden of disease and comorbidities among patients. This highlights the importance of the management of comorbidities in the optimal care of patients with NAFLD/NASH. The Japanese NAFLD/NASH patient population in this study was elderly, with almost half aged ≥ 65 years, and exhibited a high prevalence of comorbidities which generally increased with age. Accordingly, NAFLD/NASH patients incurred substantial all-cause healthcare resource use and costs.

## Supplementary Information

Below is the link to the electronic supplementary material.

Supplementary file1 (XLSX 44 KB)

Supplementary file2 (XLSX 113 KB)

Supplementary file3 (PDF 160 KB)

## Data Availability

The data sets analysed in this study can be purchased from Medical Data Vision (MDV). However, the authors cannot share the data with any third parties or make the data publicly available due to protections around the sharing of private health data.
