# Supplementary material for: Comorbidities and healthcare costs and resource use of patients with nonalcoholic fatty liver disease (NAFLD) and nonalcoholic steatohepatitis (NASH) in the Japan medical data vision database
Source: J Gastroenterol. 2021 Jan 26;56(3):274–84. doi: 10.1007/s00535-021-01759-2 (PMC7932941; doi:10.1007/s00535-021-01759-2)
Supplement: Supplementary file 3 — Supplementary file3 (PDF 160 KB) [file 535_2021_1759_MOESM3_ESM.pdf]

SUPPLEMENTARY MATERIALS

Comorbidities and Healthcare Costs and Resource Use of Patients with Nonalcoholic Fatty Liver Disease (NAFLD) and Nonalcoholic Steatohepatitis (NASH) in the Japan Medical Data Vision Database

Shuji Terai[1], Amy Buchanan-Hughes[2], Alvin Ng[3], I-Heng Lee[4], Ken Hasegawa[4]

[1]Division of Gastroenterology and Hepatology, Graduate School of Medical and Dental Sciences, Niigata University, Niigata, Japan;

[2]Costello Medical, Boston, MA, USA;

[3]Costello Medical, Singapore, Singapore;

[4]Gilead Sciences Inc, Foster City, CA, USA

Correspondence to: Ken Hasegawa (ken.hasegawa1@gilead.com)

Journal: Journal of Gastroenterology

Supplementary Table 1 Demographic characteristics of overall population, and stratified by year of index date. Patients who had their index dates in each particular year were included in that year's analysis

Variable		At index date (N = 58,958) n (%)	P value[a]	2011 and pre-2011 (N = 11,515) n (%)	2012 (N = 3,310) n (%)	2013 (N = 4,629) n (%)	2014 (N = 7,046) n (%)	2015 (N = 10,037) n (%)	2016 (N = 11,535) n (%)	2017 (N = 10,886) n (%)	P Value[b]
Gender	Male	32,698 (55.46)	<0.001	6,429 (55.83)	1,904 (57.52)	2,575 (55.63)	3,952 (56.09)	5,578 (55.57)	6,325 (54.83)	5,935 (54.52)	0.038
	Female	26,260 (44.54)		5,086 (44.17)	1,406 (42.48)	2,054 (44.37)	3,094 (43.91)	4,459 (44.43)	5,210 (45.17)	4,951 (45.48)	
Age group, years	18—34	2,409 (4.09)	N/A	264 (2.29)	142 (4.29)	205 (4.43)	336 (4.77)	463 (4.61)	508 (4.40)	491 (4.51)	N/A
	35—44	5,293 (8.98)		823 (7.15)	330 (9.97)	502 (10.84)	680 (9.65)	956 (9.52)	1,070 (9.28)	932 (8.56)	
	45—54	8,897 (15.09)		1,529 (13.28)	498 (15.05)	675 (14.58)	1,052 (14.93)	1,599 (15.93)	1,837 (15.93)	1,707 (15.68)	
	55—64	14,334 (24.31)		3,210 (27.88)	904 (27.31)	1,181 (25.51)	1,758 (24.95)	2,351 (23.42)	2,534 (21.97)	2,396 (22.01)	
	65—74	17,434 (29.57)		3,410 (29.61)	894 (27.01)	1,318 (28.47)	2,054 (29.15)	2,953 (29.42)	3,521 (30.52)	3,284 (30.17)	
	75+	10,591 (17.96)		2,279 (19.79)	542 (16.37)	748 (16.16)	1,166 (16.55)	1,715 (17.09)	2,065 (17.90)	2,076 (19.07)	
Age, years	Mean	61.63		63.29	60.76	60.75	60.91	61.01	61.41	61.77	<0.001
	SD	13.99		12.93	13.86	14.09	14.23	14.23	14.21	14.30	
	Median	64.00		64.00	63.00	63.00	63.00	63.00	64.00	64.00	

[a]One-way chi-squared test with expected frequencies of gender as reported in 2015 Japanese Census. [b]Pearson's chi-squared tests of association between gender and year of enrolment from 2012 to 2017; comparisons of mean age across years from 2012 to 2017 with ANOVA. *SD* standard deviation

Supplementary Table 2 Demographic characteristics of patients in the NASH subgroup. NASH subgroup includes patients (with or without NAFLD) with at least one claim for NASH at any time during the study; in the NASH subgroup, the index date was the date of the first claim for either NASH or NAFLD

Variable		NASH (N = 1,139) n (%)
Gender	Male	574 (50.4)
	Female	565 (49.6)
Age group	18-34 years	73 (6.4)
	35-44 years	125 (11.0)
	45-54 years	209 (18.4)
	55-64 years	229 (20.1)
	65-74 years	286 (25.1)
	75+ years	217 (19.1)
Age, years	Mean (SD)	59.87 (15.56)
	Median (IQR)	62.00 (23.00)

IQR interquartile range, *NAFLD* nonalcoholic fatty liver disease, *NASH* nonalcoholic steatohepatitis, *SD* standard deviation

Supplementary Table 3 BMI categories for patients with available height and weight data. Height and weight data were only available for patients who had been hospitalized. BMI categories were determined based on Asia-Pacific obesity classification criteria

BMI category	All	Ages 18 to 34	Ages 35 to 44	Ages 45 to 54	Ages 55 to 64	Ages 65 to 74	Ages 75 and above
	(N = 9,620)	(N = 368)	(N = 730)	(N = 1,169)	(N = 1,972)	(N = 2,970)	(N = 2,411)
	n (%)	n (%)	n (%)	n (%)	n (%)	n (%)	n (%)
Underweight (BMI<18.5)	315 (3.27)	7 (1.90)	13 (1.78)	29 (2.48)	33 (1.67)	84 (2.83)	149 (6.18)
Normal weight (18.5≤BMI<23.0)	2,131 (22.15)	51 (13.86)	86 (11.78)	160 (13.69)	400 (20.28)	724 (24.38)	710 (29.45)
Overweight/"at risk" (23.0≤BMI<25.0)	1,864 (19.38)	34 (9.24)	63 (8.63)	171 (14.63)	386 (19.57)	658 (22.15)	552 (22.90)
Obese I (25.0≤BMI<30.0)	3,658 (38.02)	105 (28.53)	288 (39.45)	477 (40.80)	821 (41.63)	1,155 (38.89)	812 (33.68)
Obese II (BMI≥30)	1,652 (17.17)	171 (46.47)	280 (38.36)	332 (28.40)	332 (16.84)	349 (11.75)	188 (7.80)

BMI body mass index.

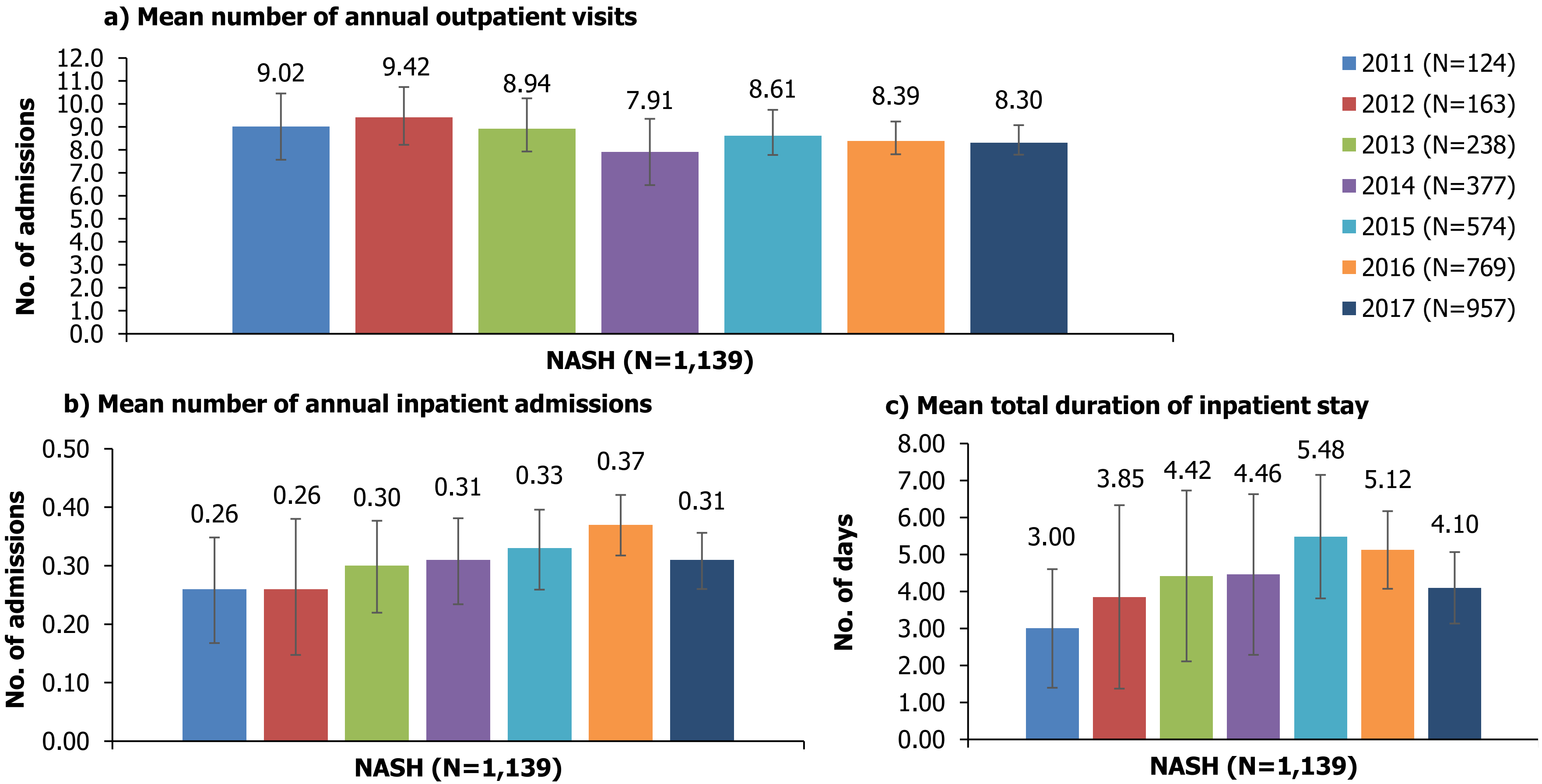

Supplementary Fig. 1 Inpatient resource use for patients in the NASH subgroup. NASH subgroup includes patients (with or without NAFLD) with at least one claim for NASH at any time during the study. Total duration of inpatient stay is total days per year, not length of stay per admission. Error bars represent 95% confidence intervals. *NASH* nonalcoholic steatohepatitis

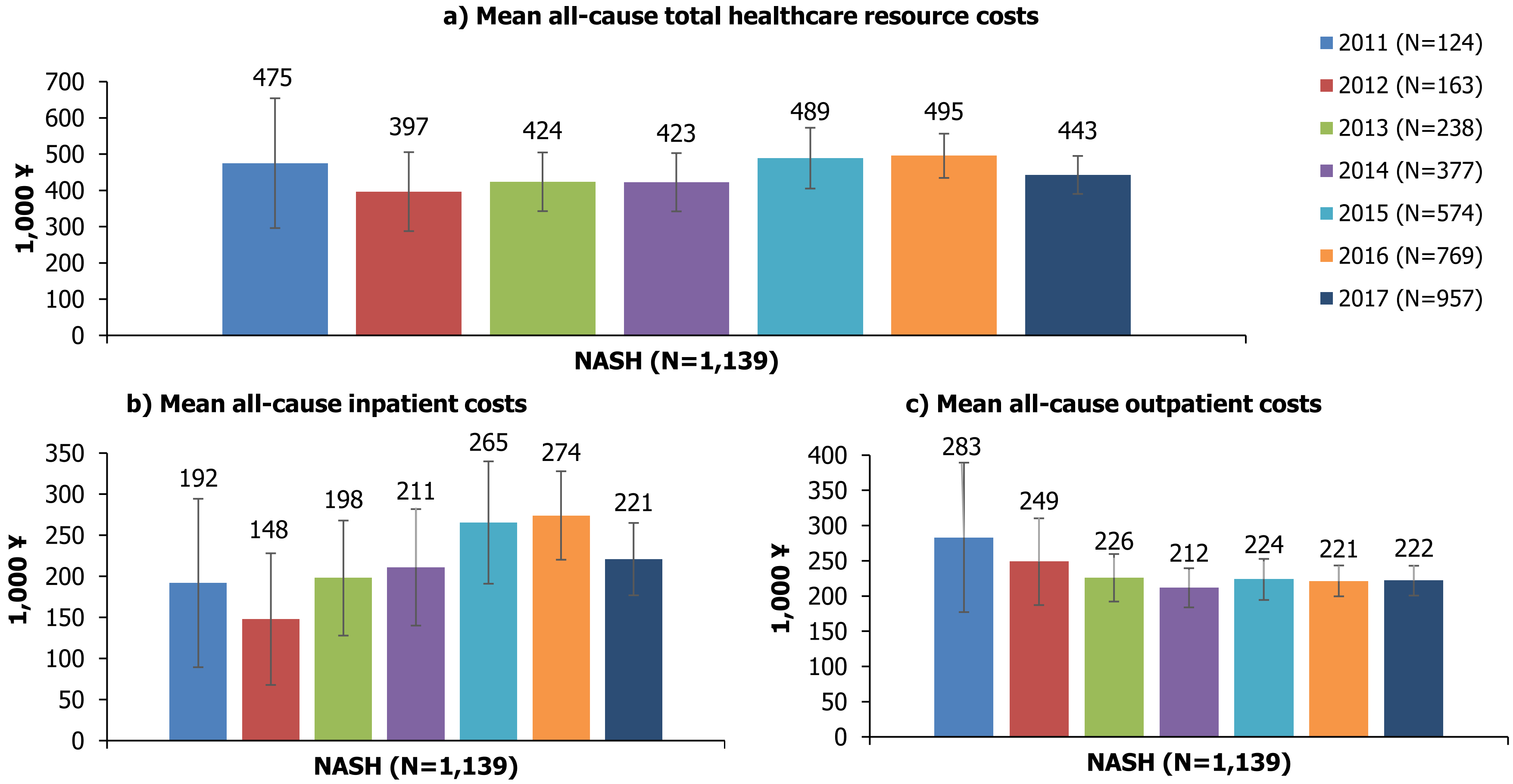

Supplementary Fig. 2 All-cause healthcare resource costs for patients in the NASH subgroup. NASH subgroup includes patients (with or without NAFLD) with at least one claim for NASH at any time during the study. Error bars represent 95% confidence intervals. *NASH* nonalcoholic steatohepatitis
